# Supplementary material for: Theoretical modeling of dendrite growth from conductive wire electro-polymerization
Source: Sci Rep. 2022 Apr 16;12:6395. doi: 10.1038/s41598-022-10082-6 (PMC9013362; doi:10.1038/s41598-022-10082-6)
Supplement: Supplementary file 1 — Supplementary Video Legends. [file 41598_2022_10082_MOESM1_ESM.pdf]

Legends of supplementary videos:

Supplementary Video S1.mp4

Video of the simulation studies representing the dendritic growth at signal-frequency of 2.5 f_o (low frequency).

Supplementary Video S2.mp4

Video of experimental studies representing the dendritic growth at signal-frequency of 20 Hz (low frequency).

Supplementary Video S3.mp4

Video of simulation studies representing the dendritic growth at signal-frequency of 25 f_o (high frequency).

Supplementary Video S4.mp4

Video of experimental studies representing the dendritic growth at signal-frequency of 850 Hz (high frequency).
