# Supplementary material for: Theoretical modeling of dendrite growth from conductive wire electro-polymerization
Source: Sci Rep. 2022 Apr 16;12:6395. doi: 10.1038/s41598-022-10082-6 (PMC9013362; doi:10.1038/s41598-022-10082-6)
Supplement: Supplementary file 6 — Supplementary Figure S1. [file 41598_2022_10082_MOESM6_ESM.pdf]

Theoretical modeling of dendrite growth from conductive wire electro-polymerization

Ankush Kumar[1]*, Kamila Janzakova[1], Yannick Coffinier[1], Sébastien Pecqueur[1], Fabien Alibart[1,2]

1 Univ. Lille, CNRS, Centrale Lille, Univ. Polytechnique Hauts-de-France, UMR 8520 - IEMN, F59000 Lille, France.

2 Laboratoire Nanotechnologies &Nanosystèmes (LN2), CNRS, Université de Sherbrooke, J1X0A5, Sherbrooke, Canada.

*ankush.kumar@iemn.fr, ankush.science@gmail.com

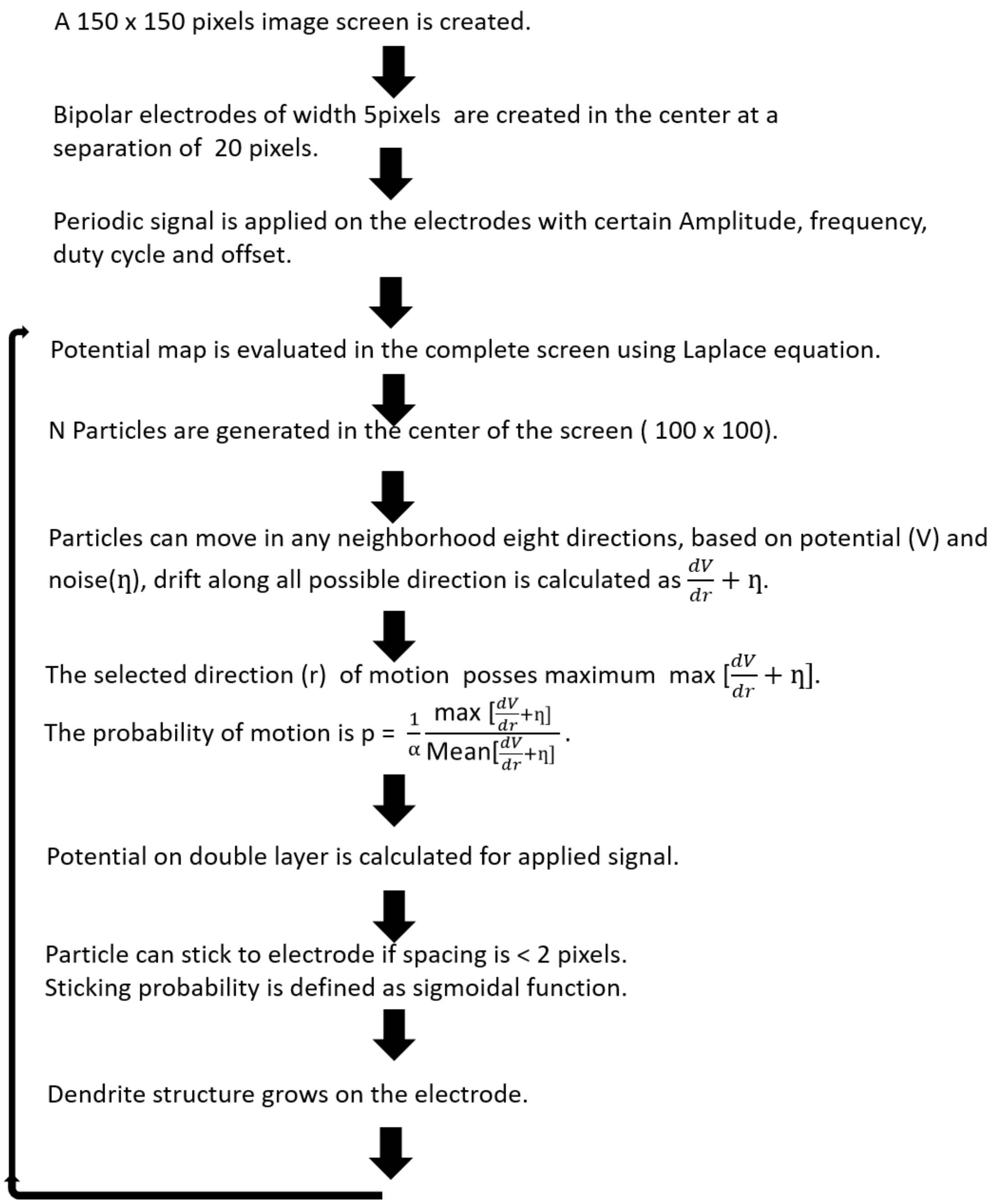

Figure S1: Simulation steps for modeling of dendrite growth from conductive wire electro-polymerization
